# Accurate flexible refinement of atomic models against medium-resolution cryo-EM maps using damped dynamics

**DOI:** 10.1186/s12900-018-0089-0

**Published:** 2018-09-15

**Authors:** Julio A. Kovacs, Vitold E. Galkin, Willy Wriggers

**Affiliations:** 1 Department of Mechanical and Aerospace Engineering, Old Dominion University, Norfolk, VA USA; 2 Department of Physiological Sciences, Eastern Virginia Medical School, Norfolk, VA USA

**Keywords:** Electron-microscopy map, Density fitting, Conformational change, Protein flexibility, Damped dynamics

## Abstract

**Background:**

Dramatic progress has recently been made in cryo-electron microscopy technologies, which now make possible the reconstruction of a growing number of biomolecular structures to near-atomic resolution. However, the need persists for fitting and refinement approaches that address those cases that require modeling assistance.

**Methods:**

In this paper, we describe algorithms to optimize the performance of such medium-resolution refinement methods. These algorithms aim to automatically optimize the parameters that define the density shape of the flexibly fitted model, as well as the time-dependent damper cutoff distance. Atomic distance constraints can be prescribed for cases where extra containment of parts of the structure is helpful, such as in regions where the density map is poorly defined. Also, we propose a simple stopping criterion that estimates the probable onset of overfitting during the simulation.

**Results:**

The new set of algorithms produce more accurate fitting and refinement results, and yield a faster rate of convergence of the trajectory toward the fitted conformation. The latter is also more reliable due to the overfitting warning provided to the user.

**Conclusions:**

The algorithms described here were implemented in the new Damped-Dynamics Flexible Fitting simulation tool “DDforge” in the *Situs* package.

**Electronic supplementary material:**

The online version of this article (10.1186/s12900-018-0089-0) contains supplementary material, which is available to authorized users.

## Background

Over the last few years, experimental techniques for cryo-electron microscopy (cryo-EM) have evolved dramatically, making it possible for some structures to be solved at near-atomic resolution (See e.g. refs. [1–4] for reviews.). However, a significant number of structures still defy EM reconstructions at that resolution level. Also, during a typical cryo-EM workflow, resolution normally increases over time, from a rather low value, as the quality and quantity of the images improves. These considerations indicate that there is still a need to be able to obtain atomic models from cryo-EM maps having medium resolutions, in the range of 5–10 Å.

The original “Damped-Dynamics Flexible Fitting” (DDFF) simulation engine [5], on which we base our present development, predates the revolutionary improvement in cryo-EM techniques, and was targeted mainly to low-resolution EM maps. Several new flexible-fitting approaches have been published since then, which can be broadly grouped into a few types:

- Methods based on elastic network models [6–10].
- Methods based on molecular-dynamics simulations [11–14].
- Methods based on graph-theoretic approaches to determine correspondences between secondary-structure elements [15–17].
- Others:
  - Force field heuristically defined in terms of the “overlap” between the EM map and the model [18];
  - Monte-Carlo optimization of fragments combined with all-atom refinement [19];
  - Bayesian-based refinement [20].

Despite the undeniable successes of these methods, they still suffer from one or more weaknesses, including: high complexity (time and algorithmic), potentially insufficient sampling of the conformational space, restrictions in the degrees of freedom (DOFs) (such as using rigid domains), requirement of significant user intervention, and even unnaturalness of the resulting deformations.

DDFF attempts to address these issues by using an idea quite different from those of the other proposals: damped dynamics [5]. Damped dynamics operates in generalized coordinates (positional and internal coordinates) and avoids harmonic potentials by using dampers (shock absorbers) between pairs of atoms.

One of the key improvements proposed in the present paper over the original DDFF is particularly beneficial to the medium-resolution range (5 to 10 Å): the optimization of parameters used in the computation of the model-induced map. These parameters are the width of the Gaussian kernel with which the atomic model is convolved, and the density threshold value to be used after convolution. Together, the new methods yield an accurate force field that attracts the atomic model to unoccupied regions of the EM map.

A second new feature that is unique to the current “DDforge” implementation described below is the ability for the user to impose distance constraints between specified pairs of pseudo-atoms. We show an example of this to help preserve the overall shape of *β*-sheets.

A third new feature is a streamlined scheme to continuously adjust the cutoff distance *d*_*cut*_ between pseudo-atoms to be connected by dampers. The new scheme is the result of a rationale that yields an updated *d*_*cut*_ at each time step, in terms of the previous one and the ratio of the previous and current RMS velocities. As a result, the convergence rate of the DDforge trajectories is significantly improved.

Lastly, we propose a simple mechanism to guard against overfitting. This consists in estimating characteristic saturation times of the overlap function via a continually updated exponential regression.

## Methods

Our force field relies on a simulated model-induced map that is matched with the experimental EM map. After a preliminary rigid-body fit, the force field is applied to the damped dynamics of coarse-grained side chains to guide the atomic model towards unoccupied regions of the experimental target map [5]. The matching of the model-induced map with the experimental map is therefore an important aspect in the accurate refinement of the structure.

### Optimization of parameters for the model-induced map

**Fig. 1:**
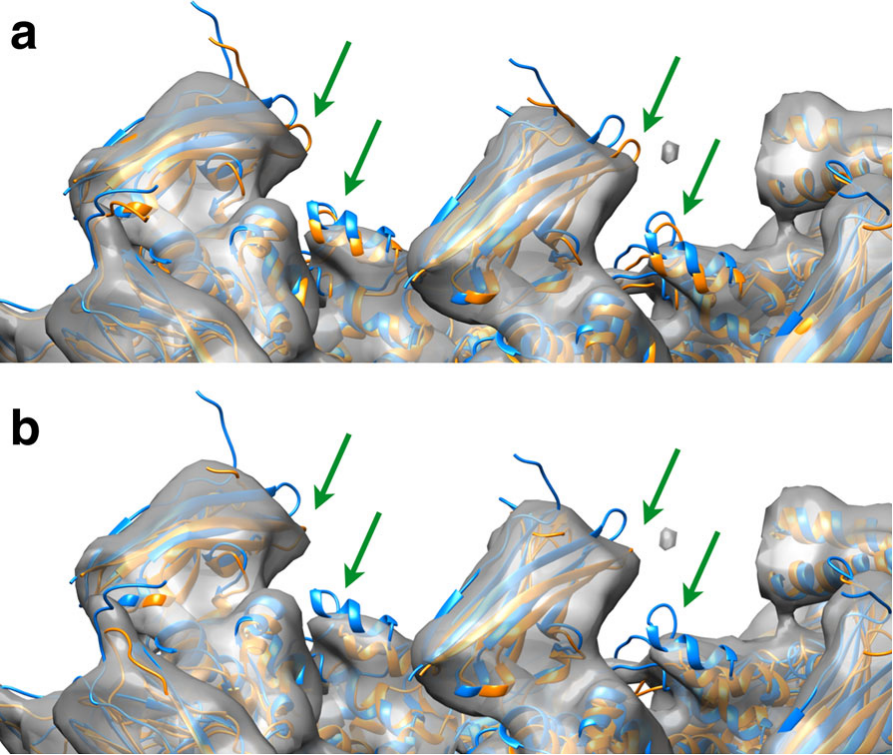
Comparison between fitting results obtained with DDFF (**a**) i.e., using $\sigma =R/(2\sqrt {3})$ and *T* determined by equating volumes at mean values, with those obtained with DDforge (**b**) whereby *σ* and *T* are simultaneously optimized by following a procedure depicted in Fig. 2

The original DDFF approach used a fixed standard deviation *σ* of the Gaussian convolution kernel based on the *Situs* convention: $\sigma =R/(2\sqrt {3})$ (section 4 in ref. [21]), where *R* is the nominal resolution of an EM map as determined by the experimentalists. This fixed *σ* convention was reasonable for the interpretation of low-resolution maps with the older Situs tools. However, for intermediate resolution maps using the new DDforge, we found it beneficial to allow *σ* to be a free parameter that can be optimized from an initial guess such as $\sigma =R/(2\sqrt {3})$. The optimization of *σ* can be shown in some cases to provide more accurate fitting results (Fig. 1), and it adds only one additional free parameter, which is negligible compared to the many conformational DOFs in the damped dynamics model.

**Fig. 2:**
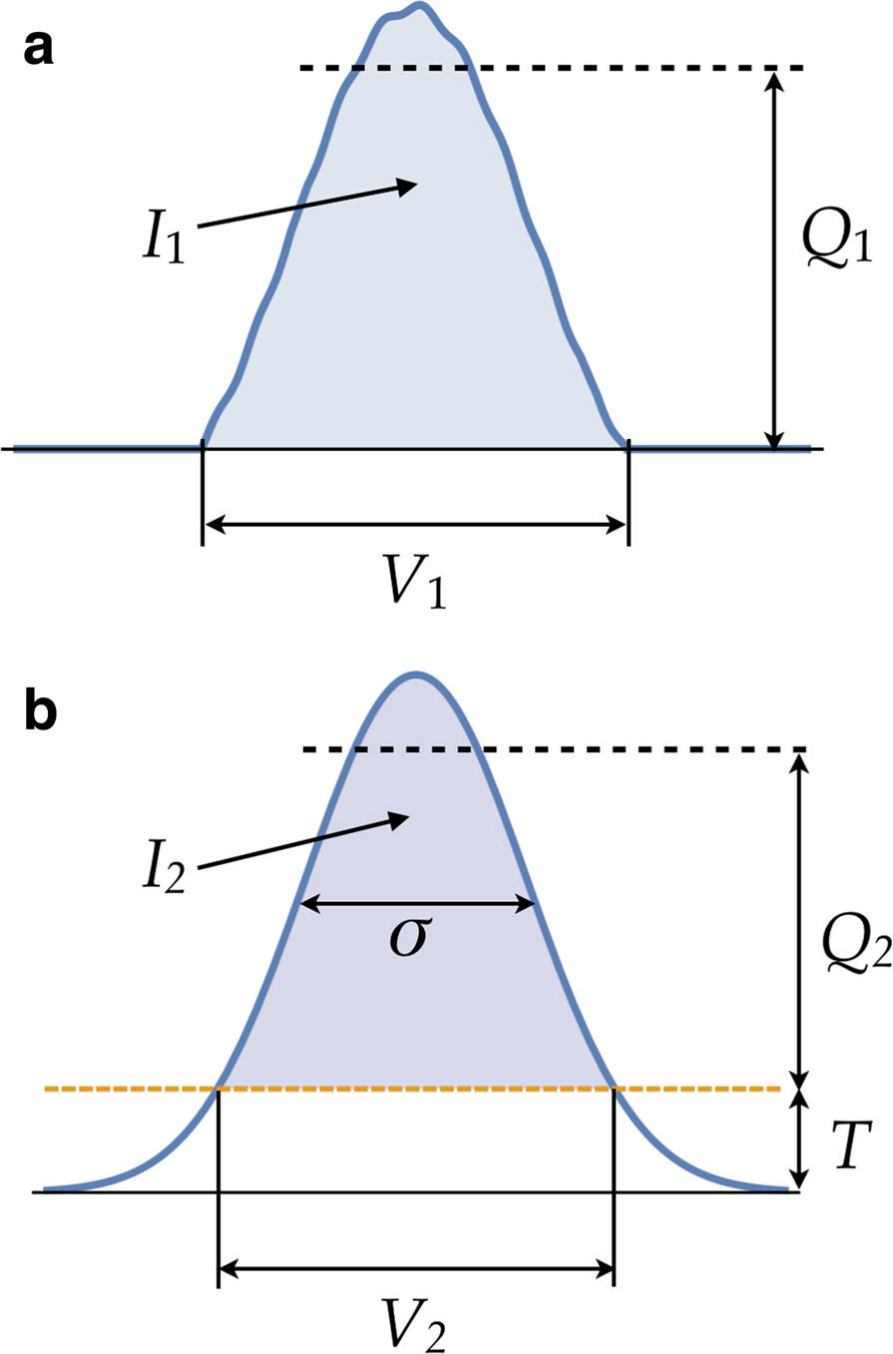
Schematic depiction of the scheme used for finding the optimal values of kernel width *σ* and threshold *T*. **a** EM map (after it has been thresholded) from which its volume *V*_1_, integral *I*_1_, and 95% quantile value *Q*_1_ are computed. **b** Model-induced map (before thresholding), with the corresponding quantities *V*_2_, *I*_2_ and *Q*_2_, in addition to the parameters *σ* and *T*. These parameters are determined as those for which *V*, *I* and *Q* of both maps match, after proper scaling between the two maps

A second parameter which we optimize in the new DDforge is the threshold *T* to be applied after Gaussian kernel convolution. In the original DDFF engine, we implemented two options to determine the threshold *T*: (a) so that the volume within the isosurface of mean value matches the corresponding volume in the EM map; *or* (b) so that, after rescaling, the integral and maximum coincide with the respective values for the EM map. In the new approach, we merge these two alternatives into one. In this way, by considering *σ* as an extra unknown, we can have *all three* quantities match: volume, integral, and maximum. Actually, the maximum density (which is sensitive to outliers) was replaced by a more stable value: the 95% quantile. Likewise, the mean value for the isosurface was replaced by the *threshold*.

Figure 2 provides an overview of the density matching and optimization steps. In summary, we have the following system of equations in the new DDforge:

1 $$\begin{array}{*{20}l} V_{2} &= V_{1},  \end{array} $$

2 $$\begin{array}{*{20}l} a I_{2} &= I_{1},  \end{array} $$

3 $$\begin{array}{*{20}l} a Q_{2} &= Q_{1},  \end{array} $$

where *V* denotes the volume, *I* the integral, and *Q* the 95% quantile, subindex 1 corresponding to the EM map and subindex 2 to the model-induced map. The quantities on the right side of the equations are computed directly from the EM map. The scaling factor *a* is to be solved for as well, along with *σ* and *T*. Note that *a* does not enter into the first equation since the volume does not depend on the scaling.

We solve these equations as follows. Let *g* denote the model-induced map, computed as the convolution of the atomic model with a Gaussian kernel of standard deviation *σ*. This map, like the EM map, is defined on a set $\mathcal {R}$ of *N* voxels. In what follows, we denote a generic voxel (or its coordinates) by *x*. Then the integral *I*_2_ is given by

4 $$ I_{2} = \sum_{g(x)>T}(g(x)-T),  $$

so from Eq. 2 we obtain:

5 $$ a \sum_{g(x)>T}(g(x)-T) = I_{1}.  $$

The volumes in Eq. 1 are conveniently expressed as number of voxels. Thus,

6 $$ V_{2} = \#\{ x\in\mathcal{R} \:\vert\: g(x)>T \}.  $$

If we let $\bar {g}$ denote the 1D array of *N* values of *g* sorted in ascending order, i.e., $\bar {g}_{1} \leq \bar {g}_{2} \leq \dots \leq \bar {g}_{N}$, then Eq. 6 implies that $T = \bar {g}_{N-V_{2}}$, which together with Eq. 1 gives:

7 $$ T = \bar{g}_{N-V_{1}}.  $$

On the other hand, from Eqs. 3 and 5 we obtain:

8 $$ D(\sigma, T) \equiv \frac{\sum_{g(x)>T}(g(x)-T)}{Q_{2}}-\frac{I_{1}}{Q_{1}} = 0,  $$

where *Q*_2_ depends on *σ* and *T*, while *g* depends only on *σ*. We now have two equations, 7 and 8, in the two unknowns *σ* and *T*. The algorithm to solve them proceeds iteratively between these two equations: for each value of *σ* (starting, for instance, with $\sigma _{0}=R/(2\sqrt {3})$), build the model-induced map *g*, and compute *T* using Eq. 7. With this *T*, go to Eq. 8 and make one Newton-Raphson step on *σ*. With this updated *σ*, go back to Eq. 7 to get a new value of *T*, and continue in this way till convergence. In our examples, the number of iterations needed was very small (5 or 6), and the compute time was about a second, even for the largest structures that we considered.

### Distance constraints

Constraints are given by general relations among the generalized coordinates of the form:

9 $$ f_{\alpha}(q_{1},\dots,q_{M}) = 0 \qquad (\alpha=1,\dots,K).  $$

The way to insert these conditions into the equations of motion is by taking their time derivative:

10 $$ \sum\limits_{i=1}^{M} \frac{\partial f_{\alpha}}{\partial q_{i}} \dot{q_{i}} = 0 \qquad \forall\alpha,  $$

whence the equations of motion become:

11 $$ \left. \begin{array}{lll} \sum\limits_{i} (B_{ij}+V_{ij}) \dot{q}_{i} - \sum\limits_{\alpha} \frac{\partial f_{\alpha}}{\partial q_{j}} h_{\alpha} &= Q^{(m)}_{j} \qquad \qquad &\forall j \\ \quad \quad \sum\limits_{i} \frac{\partial f_{\alpha}}{\partial q_{i}} \dot{q}_{i} &= 0 & \forall \alpha \end{array} \right\}  $$

where the *h*_*α*_ are Lagrange multipliers and $Q^{(m)}_{j}$ is the force field in generalized coordinates.

In the particular case of *distance* constraints, the *f*_*α*_ take the form:

12 $$ f_{\alpha} = \frac{1}{2} \left\| {\mathbf{{r}}_{a} - \mathbf{{r}}_{b}} \right\|^{2},  $$

where *a* and *b* depend on *α*, **r**_*a*_ and **r**_*b*_ denoting the positions of two specific atoms whose distance we want to require to be constant throughout the trajectory. The derivatives are:

13 $$ \frac{\partial f_{\alpha}}{\partial q_{i}} = \left\langle \mathbf{{r}}_{a}-\mathbf{{r}}_{b}, \frac{\partial\mathbf{{r}}_{a}}{\partial q_{i}} - \frac{\partial\mathbf{{r}}_{b}}{\partial q_{i}} \right\rangle,  $$

where the derivatives $\frac {\partial \mathbf {{r}}_{k}}{\partial q_{i}}$ constitute the entries of the so-called *Wilson’s matrix* [22], and can be computed directly from the geometry of the current conformation of the model.

### Scheme to optimize the dampers’ cutoff distance

This parameter *d*_cut_ is the maximum distance between atoms that will be connected by a damper. The rationale for its optimization is based on the RMS velocity of the structure, defined by

14 $$ v^{2} = \frac{1}{A} \sum\limits_{i=1}^{A} \| {\dot{\mathbf{{r}}}_{i}} \|^{2},  $$

where *A* is the number of pseudo-atoms in the structure.

The idea is to adjust *d*_cut_ in such a way as to try to keep *v* constant. As the trajectory progresses, this will make *d*_cut_ gradually decrease. This decrease is allowed until *d*_cut_ reaches max{7Å,2*σ*} (which ensures a minimum of connectivity to preserve the structural integrity of the model), after which point it is kept constant, with the ensuing decrease of *v*.

To keep *v* nearly constant as much as possible, we need an estimate of the dependence of *v* on *d*_cut_. For this, we can imagine a toy model involving a collective map force *F*^(*m*)^ and a collective dampers’ constant *C* (which depends on *d*_cut_), so we have, roughly:

15 $$ v \approx \frac{F^{(m)}}{C\left(d_{\text{cut}}\right)}.  $$

The number of connections of length up to *d*_cut_ is of the order of $d_{\text {cut}}^{3}$. They can be partitioned into those of length between *r* and *r*+*dr*, for *r* ranging from to 0 to *d*_cut_, of which the number is of the order of *r*^2^ *dr*. A damper of length *r* has a strength $\sim 1/\sqrt {r}$ (Eq. 8 in ref. [5]). Therefore, the aggregate strength of all dampers up to length *d*_cut_ will be:

16 $$ C\left(d_{\text{cut}}\right) \sim \int_{0}^{d_{\text{cut}}} \frac{r^{2}}{\sqrt{r}}\,dr \sim d_{\text{cut}}^{5/2}.  $$

When *v* decreases due to a decrease in *F*^(*m*)^, we want to decrease *d*_cut_ to try to restore *v* to its former value. Thus, if we have, for the previous and current time steps:

17 $$ {} v(t-1) = \frac{F^{(m)}(t-1)}{d_{\text{cut}}(t-1)^{5/2}} \qquad \text{and} \qquad v(t) = \frac{F^{(m)}(t)}{d_{\text{cut}}(t)^{5/2}},  $$

the goal is to get *v*(*t*+1)=*v*(*t*−1) assuming *F*^(*m*)^(*t*+1)=*F*^(*m*)^(*t*). This gives

18 $$ v(t-1) = v(t+1) = \frac{F^{(m)}(t+1)}{d_{\text{cut}}(t+1)^{5/2}} = \frac{F^{(m)}(t)}{d_{\text{cut}}(t+1)^{5/2}}.  $$

Hence, if we put

19 $$ \rho = \frac{v(t)}{v(t-1)},  $$

then

20 $$ \rho = \frac{F^{(m)}(t)}{d_{\text{cut}}(t)^{5/2}} \cdot \frac{d_{\text{cut}}(t+1)^{5/2}}{F^{(m)}(t)} = \left(\frac{d_{\text{cut}}(t+1)}{d_{\text{cut}}(t)} \right)^{5/2},  $$

from where we get

21 $$ d_{\text{cut}}(t+1) = d_{\text{cut}}(t) \cdot \rho^{2/5}.  $$

This is the optimal way to update *d*_cut_ based on the ratio *ρ* of the current and previous velocities.

### Stopping criterion

Overfitting is always a concern in flexible-fitting methods. In general, it is difficult to give an objective criterion in this regard. However, the fact that our method furnishes a whole trajectory of conformations—rather than a single one—provides us with a way to determine a “safe” final conformation. We do this by resorting to the plot of the overlap evolution, shown in the figures for each of our examples. The idea is that the start of the “saturation” of the graph can be considered as a warning time, after which any additional refinement is likely to lead to overfitting.

A concrete rule of thumb to estimate such a critical point in the trajectory is as follows. An exponential function, of the form

22 $$ y(t) = b-c \exp(-kt)  $$

is least-squares fitted to the graph of the overlap function. (Note that in this paper the variable *t* denotes the time step number rather than a physical time.) This is a non-linear exponential regression problem on *b*,*c*,*k*, which is solved by first eliminating the linear parameters *b* and *c* from the regression equations, and then numerically solving the resulting non-linear equation for *k*. With these parameters, we can define the *warning time* as the time step *t*_1_ such that the exponential function above reaches a fraction *α*_1_ of the interval between *y*(0)=*b*−*c* and *y*(*∞*)=*b*. This gives

23 $$ t_{1} = \frac{1}{k} \ln{\frac{1}{1-\alpha_{1}}}.  $$

Since the trajectory is being obtained dynamically, the above process needs to be performed at each time step, using the points of the overlap function that have been computed until the current step. Thus, as the simulation proceeds, updated values of the regression parameters are obtained, and so we need to carry on the simulation sufficiently long, to be certain that we have enough points to yield an accurate fit. Updated estimates of this *stopping time* *t*_2_ are obtained in a manner similar to *t*_1_: by using a value *α*_2_ instead of *α*_1_:

24 $$ t_{2} = \frac{1}{k} \ln{\frac{1}{1-\alpha_{2}}}.  $$

**Fig. 3:**
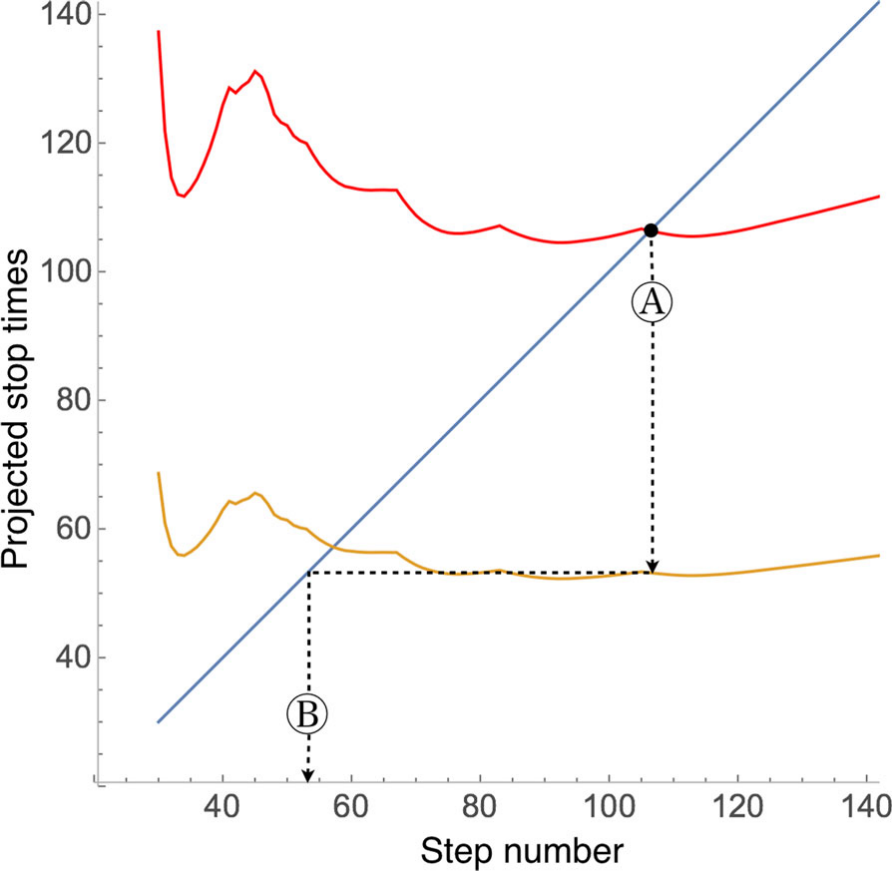
Determination of the times for stopping the simulation and of the possible onset of overfitting. The simulation proceeds until point A, where the estimate of the *stopping time* (red curve) becomes equal to the current time (blue line). At that point, the estimate of the onset of overfitting is given by the orange curve (point B: *warning time*). (This plot corresponds to the lactoferrin test case. See Fig. 4, where the value *t*_1_=53 (warning time, see “Methods” Section) obtained from this plot is indicated.)

The simulation then proceeds as long as the estimate *t*_2_ is bigger than the current time step. When the point is reached at which this condition is no longer satisfied, the simulation is stopped and the current estimate of *t*_1_ is defined as the *warning time*, after which point overfitting is likely to begin. This process is described graphically in Fig. 3 (which is the actual plot for the lactoferrin test case considered below). Adequate values that worked well in our examples are *α*_1_=0.9 and *α*_2_=0.99.

**Fig. 4:**
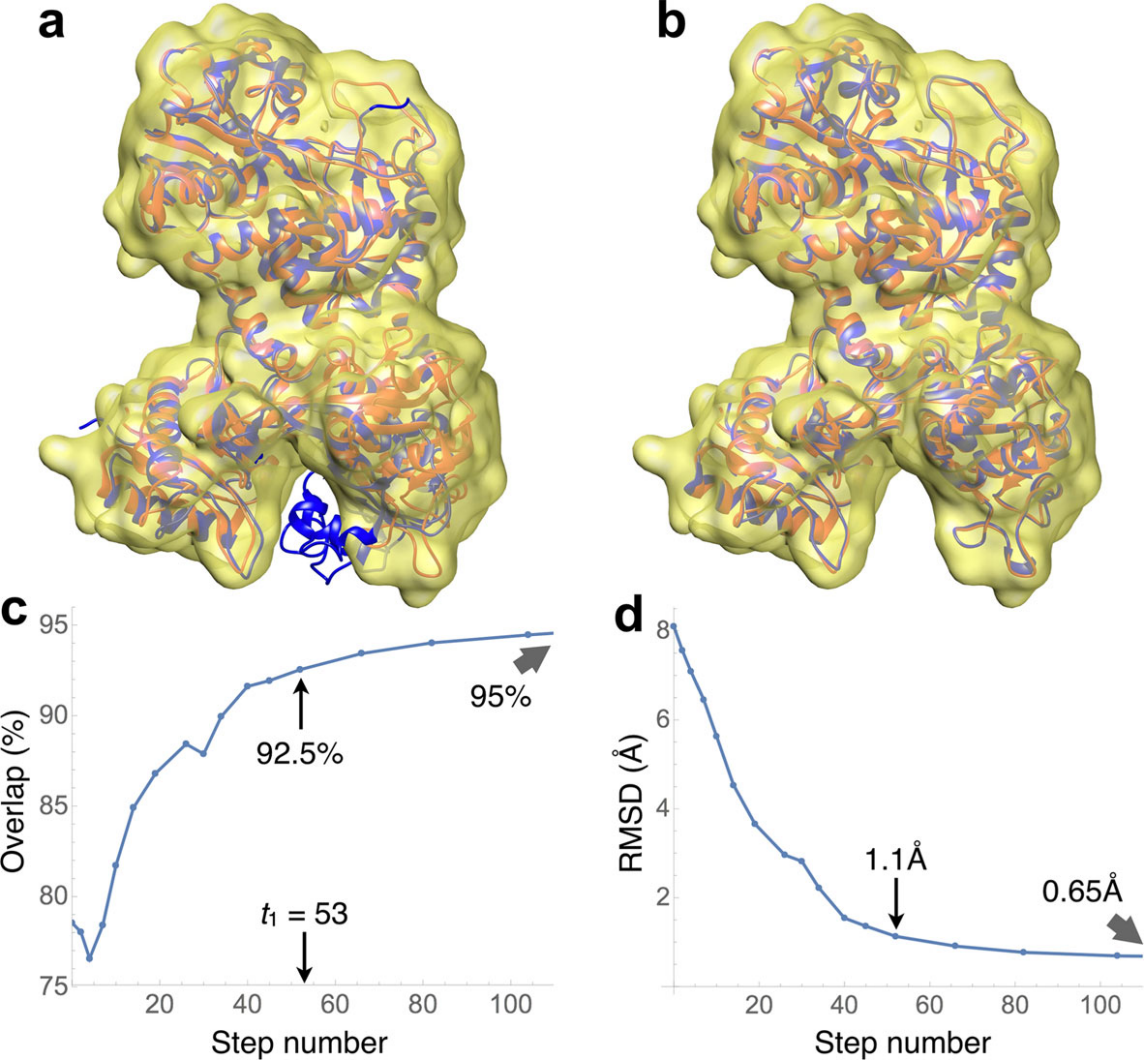
Fully flexible fitting of the closed (iron-bound) conformation of lactoferrin (PDB code 1lfg) into a simulated density map (yellow surface) at 7Å resolution. Shown in red is the open conformation (apolactoferrin, PDB code 1lfh), which was used to generate the map. **a** The closed conformation is shown in blue. This was the starting conformation used for the fitting. **b** The final conformation of the trajectory is displayed in blue. **c** Evolution of the overlap values along the trajectory. The indicated value of *t*_1_=53 is the warning time, where overfitting is likely to begin. At that time, the overlap was 92.5% **d** Evolution of the backbone RMSD values along the trajectory. The RMSD was 1.1Å at the *t*_1_ time. The stopping time for this case was *t*_2_=107

### Side-chain optimization

DDforge includes an option to optimize the side-chain conformations along the trajectory. This step can help to escape from wrong side-chain geometries that could occur if they were simply evolved from their initial conformations, and it also compensates for the inaccuracy introduced by the reduced-residue model. The side-chain optimization (which in our examples was done for each conformation written out to disk) is performed by the SCWRL4 method [23], which uses an efficient tree-decomposition algorithm that furnishes the best side-chain conformations for each given backbone geometry by minimizing a simplified atomic force field on a rotamer library.

## Results

We describe several simulated and experimental cases with the main purpose of illustrating the advantage of the new features introduced in the present work. Side-chain optimization was used in all cases except thermosome, since in this case the focus was on modeling the considerable large-scale deformations between the atomic model and the EM map.

### Validation test: lactoferrin

To demonstrate the level of accuracy achievable by means of the new features described in the previous section, we consider the simulated case of lactoferrin. This is an iron-binding protein that undergoes a large conformational change of about 8Å RMSD where three rigid domains rotate about hinge axes. Due to the piecewise-rigid motion, the system is a standard for testing rigid-body modeling techniques [24], but the highly localized flexibility—present only in the hinge regions between rigid domains—creates a formidable challenge for many flexible refinement methods.

The simulated maps were generated from the apolactoferrin structure ([25], PDB code 1lfh), and the atomic model that was fitted into those maps was the iron-bound structure ([26], PDB code 1lfg). These structures have 691 residues. See Fig. 4a and b. This experiment consisted of four cases:

- Simulated map to 7Å resolution;
- Simulated map to 15Å resolution;

each combined with:

- Atomic model fully flexible;
- Atomic model with helices and *β*-strands rigid.

In the fully flexible case, the number of free variables was 1888, while the number was 1266 when keeping helices and strands rigid.

Table 1 summarizes the results of this experiment and compares them with those from other methods. We see that, for all the DDforge cases, the RMSD of the final conformation from the target is more than an order of magnitude better than the resolution of the simulated map. Moreover, since the rigid domains (when superimposed) exhibit RMSD values between only 0.42 and 0.65Å [27], our flexible refinement accuracy of 0.58 and 0.65Å (in the 7Å case) is actually at the level of the residual discrepancy between the rigid domains. (The RMSDs for DDforge are measured on all backbone atoms.)

**Table 1:** RMSDs, in Å, for the flexible fitting of lactoferrin by various methods

	DDforge				
*R*	fully flexible	H+S rigid	IMF	CGS	VQ
7	0.65	0.58	—	—	—
15	1.04	0.89	0.98	1.89^a^	2.72

*R* is the resolution, in Å, of the simulated maps. Each of these RMSD values is between the fitted conformation and the target atomic structure used to create the simulated map. The RMSDs for DDforge are measured on all the backbone atoms, while those for the other methods are measured on the *α*-carbons only. H+S: helices and *β*-strands; IMF: Iterative Modular Fitting [28]; CGS: Constrained Geometric Simulations [40]; VQ: Vector Quantization without rigidity constraints [41]

^a^This actually corresponds to a 14Å map

Even though we are mostly interested in the 5–10 Å resolution range, we wanted to compare the performance of our method with the lactoferrin results reported in ref. [28], with RMSDs of 1.89Å and 2.72Å reported for two flexible fitting methods. The rigid-body Iterative Modular Fitting (IMF) method was found to surpass the flexible methods, with an RMSD of 0.98Å. Volkmann [28] concludes that 5Å resolution is needed for a flexible-fitting approach (namely MDFF [12]) to yield better results than IMF. We note from Table 1, however, that DDforge matches the accuracy of IMF at 15Å resolution. One should consider that IMF is a piecewise-rigid method for which lactoferrin’s piecewise-rigid motion is obviously well suited by design. DDforge, by contrast, is a truly flexible refinement that can handle longer-range deformations (see below), but it handles even the special case of lactoferrin’s piecewise-rigid motion well.

The runtime for this case, on a desktop computer with a 4 GHz Intel Core i7 processor and 32 GB of RAM, running on a single core, was about 9 min or 11 min, with or without side-chain optimization, respectively. These timings will vary depending on parameters used and on the number of conformations saved (since the side-chain optimization is performed for each saved conformation), so they should be taken only as a guideline.

Figure 4c and d show the evolution of the overlap and RMSD with respect to the target, for the all-flexible fitting into the 7Å map: the overlap increases from 78% to 95%, while the RMSD decreases from 8.1Å to 0.65Å. These RMSD values are caused by rigid motions of domains and by their internal flexible refinement.

### Tests with experimental EM maps

#### Thermosome

**Fig. 5:**
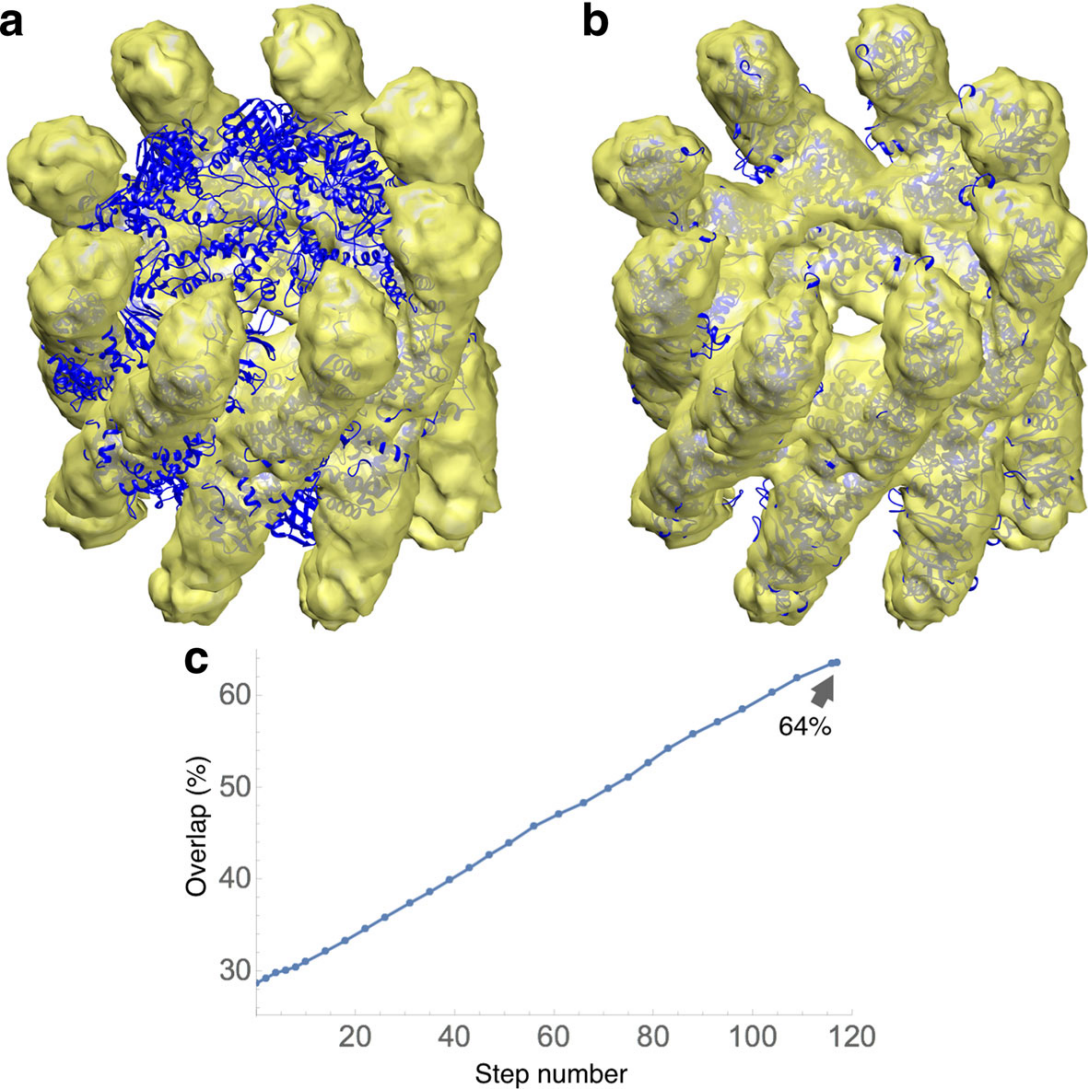
Flexible fitting of the closed conformation of thermosome hexadecamer into an EM map of the open conformation (yellow surface) to 10Å resolution. Helices and *β*-strands were kept rigid. **a** The closed conformation of thermosome is shown in blue. This was the starting conformation used for the fitting. **b** The final conformation of the trajectory is displayed in blue. **c** Evolution of the overlap values along the trajectory. In this case, the trajectory was ended much earlier than the stopping time, since the speed had a fast decay at the end of the time period displayed. Thus, the trajectory did not reach the overfitting regime

Thermosome is an archaeal chaperonin from *T. acidophilum*. The asymmetric unit of the crystal structure [29] is a dimer (PDB code 1a6d); we used the hexadecameric biological assembly with D _4_ symmetry as the atomic model for fitting (Fig. 5). This structure has a total of 8040 residues, making it the largest structure on which we tested our method. The EM map we used was a reconstruction to 10Å resolution of the open conformation of the hexadecamer [30] (EMDB code 1396).

For this case, we kept the helices and *β*-strands rigid, with which the number of free variables was 12,664. (A fully flexible model would have had 22,360 free variables.) The atomic structure was initially positioned by eye in the EM map. Figure 5c shows the evolution of the overlap with the EM map: it increases from 29% to 64%. A movie of this trajectory is available as Additional file 1. The runtime for this case was about 5 h (with no side-chain optimization).
Additional file 1: Flexible fitting of thermosome. (MOV 9378 kb)

#### Actin filament with MyBP-C bound

**Fig. 6:**
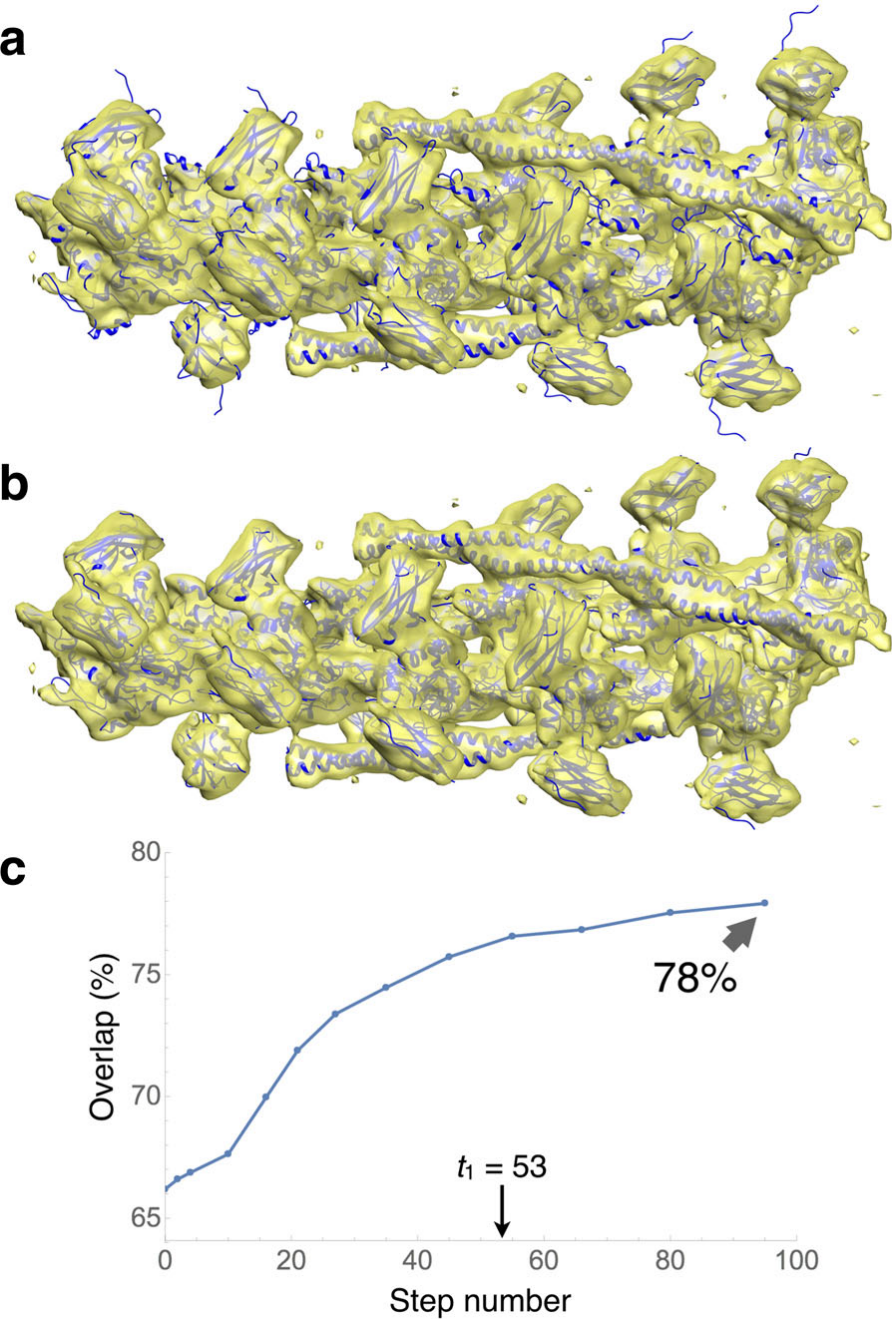
Flexible fitting of an atomic model comprised of F-actin, tropomyosin and Ig-domains of MyBP-C (blue ribbons) into an EM map (yellow surface) filtered to 11Å resolution. Helices and *β*-strands were kept rigid. In addition, distance constraints were imposed between adjacent endpoints of *β*-strands in the C0 and C1 domains. **a** The initial atomic model, docked into the electron density map using rigid body fitting, is shown in blue. **b** The resulting atomic model (final conformation of the trajectory) yielded by DDforge. **c** Evolution of the overlap values along the trajectory. The indicated value of *t*_1_=53 is the warning time, where overfitting is likely to begin. (Coincidentally, its value was equal to that for lactoferrin. See Fig. 4.) The stopping time for this case was *t*_2_=106

Immunoglobulin domains (Ig-domains) of myosin binding protein-C (MyBP-C) bind to fibrillary actin (F-actin) in a highly polymorphic fashion [31]. To evaluate how DDforge works on multiprotein complexes, we applied it to a 3D reconstruction of one of the multiple modes of binding of C0 and C1 Ig-domains of MyPB-C to F-actin complexed with tropomyosin protein (Fig. 6). The resolution of the map was determined, using the Fourier Shell Correlation (FSC) criterion of 0.143, to be 11Å. The initial atomic model—comprised of 10 actin monomers, 10 units of each C0 and C1 Ig-domains, and two pairs of tropomyosin helices—was obtained by rigid body fitting of the corresponding high-resolution structures (PDB codes 2k1m, 2v6h and 4a7f) into the EM density map.

The total number of residues in this structure was 6284. As with thermosome, helices and *β*-strands were kept rigid, making the number of free variables 12,198. Also, for this case distance constraints were imposed between adjacent endpoints of *β*-strands in the C0 and C1 domains, to help preserve their structure. The total number of such constraints was 280.

Figure 6c shows the evolution of the overlap along the trajectory, going from 66% to 78%. A movie of this trajectory is available as Additional file 2. The runtime for this case was about 10 h, including the side-chain optimization at each saved conformation.
Additional file 2: Flexible fitting of actin filament with MyBP-C bound. (MOV 5883 kb)

Figure 1 compares the fitting results described above (using the simultaneous optimization of kernel width *σ* and density threshold *T* in DDforge) with those obtained with the original DDFF which used $\sigma =R/(2\sqrt {3})$ and equated only the volumes of both maps to determine *T*. (*R* is the user-specified resolution of EM map.) The improvement of the fitting in the regions indicated by arrows is noteworthy.

#### GroEL: stability test

GroEL is another ring-like chaperonin whose tetradecameric crystal structure exhibits D _7_ symmetry (PDB code 1xck). As target EM map we used the 6Å resolution variant (EMDB code 1081). Since, for this case, the atomic structure and the map are expected to represent the same conformation, the main purpose of this test was to ascertain the stability of the DDFF approach: the fitted conformation should have, at most, a small deviation from the starting atomic structure due to any residual small differences between the crystallographic and ice-embedded EM specimens.

The total number of residues in the atomic structure was 7336. As before, helices and *β*-strands were kept rigid, with which the number of free variables was 9876. No distance constraints were imposed. The starting conformation was obtained by rigid-body fitting. The runtime for this case was 11 h (including the side-chain optimization step).

The final conformation of the trajectory was determined according to the stopping criterion described in the “Methods” Section. That final conformation had a C_*α*_ RMSD of 1.8Å relative to the starting conformation. This is entirely consistent with previous results obtained by other methods [32], providing further support to the suggestion that such an RMSD value corresponds mostly to the intrinsic discrepancy between the structures, and only marginally to any potential instability of the fitting method.

### Comparison with the original DDFF

Here we provide a brief comparison between results obtained with the new DDforge and the original DDFF. The refinement runs for the thermosome and GroEL cases actually required the new DDforge approach whose improved simulated maps (Fig. 2) enable the convergence of ring-like structures. DDFF data was available, however, for comparing the lactoferrin and actin systems mentioned above.

The original DDFF run on lactoferrin took 22 min, as compared with 9.4 min of DDforge (both done on the 7Å map, without the side-chain optimization, and with fully flexible backbone). This translates into a factor 2.34 speedup. The RMS deviation between the resulting conformations (at the same overlap level) was 0.74Å. It is also interesting to compare the RMS deviations between each of the resulting conformations (original and DDforge) and the atomic structure used to make the simulated target map: the DDforge one was 0.65Å (as indicated in Table 1), whereas the original one was 0.73Å. Naturally, it only makes sense to pay attention to such small differences when considering simulated cases such as this. In real cases such as the actin complex, the difference is much more significant (Fig. 1).

The actin timings were 9 h for DDforge versus 22 h for DDFF (without performing the side-chain optimization), which means a factor 2.4 speedup. The difference in the resulting conformations is shown in Fig. 1; the corresponding RMS deviation was 1.9Å.

## Discussion

It may be useful to recall here that DDforge works in generalized coordinates (internal coordinates—torsion angles— and global position coordinates of each chain), and thus preserves the covalent bond geometry of the structure (except for the torsions). In addition, the side chains can optionally be energy-minimized by means of the SCWRL4 method. This is generally useful in all cases (not only for high-resolution maps) mainly because it helps to resolve possible “locks” in the trajectory, and not only because we may want to look at the detailed conformation of the side chains.

The use of dampers to maintain the overall assembly of the molecule allows it to model arbitrarily large conformational changes in a natural way. A demonstration of the sensibility of the deformations generated by DDforge is provided by the lactoferrin test case above, for which previous flexible-fitting approaches would degrade the RMSD from the target conformation, relative to a rigid-body fitting of subdomains. The fact that we can obtain results comparable to the residual crystallographic discrepancy suggests that DDforge can handle both rigid and flexible refinement with high accuracy.

Another example of the ability of DDforge to handle large conformational changes in a sensible way is the thermosome. This structure has a high degree of symmetry (imposed during the map reconstruction [30]), but this symmetry was not utilized during our simulations: all the chains were treated independently of one another, and no distance constraints were imposed.

Regarding efficiency, a comparison of runtimes with the original version of the code yielded a factor 2.3 speedup for the trajectory to reach the same overlap values. This can be attributed to the new scheme to update the dampers’ cutoff distance along the trajectory. The runtimes for the new version of the code, reported earlier, vary from a few minutes to 11 h, and depend not only on the size of the structures, but also on their specific geometries and parameters that affect the speed of the trajectory. Thus, even though thermosome is the largest system, it took less compute time than GroEL and the actin complex. These timings are considerably less than benchmarks reported for the NAMD molecular dynamics program used in MDFF [33]. A comparably sized F1-ATPase system (92,224 atoms) at 100 ns simulation time would take 14–100 days of compute time even on a much more powerful 24-core machine, depending on its GPU configuration.

## Conclusions

We have developed a flexible refinement strategy termed DDforge that is well suited to handle the larger, higher-resolution maps that have become common in recent years.

Significant new features in our implementation include the simultaneous optimization of both the convolution kernel width *σ* and the density threshold *T*, by equating three quantities of the EM and synthetic maps: integral, 95% quantile, and volume; the ability for the user to define distance constraints on the model, which are useful to preserve shapes in regions where the density is not well defined; a streamlined approach to update, at every time step, the cutoff length of dampers, ensuring that the speed of the trajectory is optimal and thereby shortening the compute time; and a practical scheme to signal a point in the trajectory where overfitting is likely to start.

The application of DDforge to the recent actin data demonstrates the effect of optimizing the kernel width and density threshold to generate appropriate model maps at each step. Figure 1 contrasts the results obtained by using the original approach (fixed *σ*) with those using the optimized values. As with thermosome, the helical symmetry of this complex was not imposed in our calculations.

The stability of the DDforge approach was verified on the GroEL test case. This used a 6Å resolution EM map, and the drift from the initial to the final conformations was of only 1.8Å RMSD, which previous results suggest is mostly due to the intrinsic discrepancy between the atomic model and the EM map [32].

We should point out that our approach can be applied in other settings as well, such as transition pathways and homology/loop modeling. These involve straightforward modifications in the definition of the force field: instead of being generated by a map, the forces are defined simply to be proportional to the distance between each atom in the origin structure and the corresponding atom in the target structure.

The DDforge method will be made available in version 3.0 of the *Situs* EM fitting and interpretation package (http://situs.biomachina.org), where it fills a void for a refinement tool applicable to medium-resolution maps with discernible internal (secondary structure) features. But DDforge does not attempt to deal with atomic-resolution maps, for which other existing tools coming from crystallography are undoubtedly more suitable. Plans for future work include extending the new capability of kernel-width optimization to allow for inhomogeneous [34] and anisotropic [35, 36] resolution across the map, and to allow for inhomogeneous convolution that characterizes stability and disorder of particular side chains [37]. Likewise, we are envisioning possible ways to make our approach immune to regions of density not accounted for by the atomic model. Currently, these extra densities need to be removed prior to the simulation.

## References

[1] Bai X, McMullan G, Scheres SHW (2015). How cryo-EM is revolutionizing structural biology. Trends Biochem Sci.

[2] Nogales E, Scheres SW (2015). Cryo-EM: A unique tool for the visualization of macromolecular complexity. Mol Cell.

[3] Jonić S (2016). Cryo-electron microscopy analysis of structurally heterogeneous macromolecular complexes. Comput Struct Biotechnol J.

[4] White HE, Ignatiou A, Clare DK, Orlova EV (2017). Structural study of heterogeneous biological samples by cryoelectron microscopy and image processing. BioMed Res Int.

[5] Kovacs JA, Yeager M, Abagyan R (2008). Damped-dynamics flexible fitting. Biophys J.

[6] Schröder GF, Brunger AT, Levitt M (2007). Combining efficient conformational sampling with a deformable elastic network model facilitates structure refinement at low resolution. Structure.

[7] Gorba C, Miyashita O, Tama F (2008). Normal-mode flexible fitting of high-resolution structure of biological molecules toward one-dimensional low-resolution data. Biophys J.

[8] Zheng W (2011). Accurate flexible fitting of high-resolution protein structures into cryo-electron microscopy maps using coarse-grained pseudo-energy minimization. Biophys J.

[9] López-Blanco JR, Chacón P (2013). iMODFIT: Efficient and robust flexible fitting based on vibrational analysis in internal coordinates. J Struct Biol.

[10] López-Blanco JR, Ritchie D, Chacón P (2017). Towards a multicomponent cryo-EM density flexible fitting tool. Biophys J.

[11] Orzechowski M, Tama F (2008). Flexible fitting of high-resolution X-ray structures into cryoelectron microscopy maps using biased molecular dynamics simulations. Biophys J.

[12] Trabuco LG, Villa E, Mitra K, Frank J, Schulten K (2008). Flexible fitting of atomic structures into electron microscopy maps using molecular dynamics. Structure.

[13] Grubisic I, Shokhirev MN, Orzechowski M, Miyashita O, Tama F (2010). Biased coarse-grained molecular dynamics simulation approach for flexible fitting of X-ray structure into cryo electron microscopy maps. J Struct Biol.

[14] Miyashita O, Kobayashi C, Mori T, Sugita Y, Tama F (2017). Flexible fitting to cryo-EM density map using ensemble molecular dynamics simulations. J Comput Chem.

[15] Abeysinghe SS, Baker ML, Chiu W, Ju T (2010). Semi-isometric registration of line features for flexible fitting of protein structures. Comput Graph Forum.

[16] Dou H, Baker ML, Ju T (2015). Graph-based deformable matching of 3D line with application in protein fitting. Vis Comput.

[17] Dou H, Burrows DW, Baker ML, Ju T (2017). Flexible fitting of atomic models into cryo-EM density maps guided by helix correspondences. Biophys J.

[18] de Vries SJ, Zacharias M (2012). ATTRACT-EM: A new method for the computational assembly of large molecular machines using cryo-EM maps. PLoS ONE.

[19] DiMaio F, Song Y, Li X, Brunner MJ, Xu C, Conticello V, Egelman E, Marlovits TC, Cheng Y, Baker D (2015). Atomic-accuracy models from 4.5-Å cryo-electron microscopy data with density-guided iterative local refinement. Nat Methods.

[20] Blau C, Lindahl E (2017). All-atom ensemble refinement to cryo-EM densities with a bayesian measure of goodness-of-fit. Biophys J.

[21] Wriggers W (2012). Conventions and workflows for using *Situs*. Acta Cryst D.

[22] Wilson JEB, Decius JC, Cross PC (1955). Molecular Vibrations—The Theory of Infrared and Raman Vibrational Spectra.

[23] Krivov GG, Shapovalov MV, Dunbrack RL (2009). Improved prediction of protein side-chain conformations with SCWRL4. Proteins Struct Funct Bioinforma.

[24] Wriggers W, Schulten K (1997). Protein domain movements: Detection of rigid domains and visualization of effective rotations in comparisons of atomic coordinates. Proteins Struc Funct Genet.

[25] Norris GE, Anderson BF, Baker EN (1991). Molecular replacement solution of the structure of apolactoferrin, a protein displaying large-scale conformational change. Acta Cryst B.

[26] Haridas M, Anderson BF, Baker EN (1995). Structure of human diferric lactoferrin refined at 2.2 Å resolution. Acta Cryst D.

[27] Anderson BF, Baker HM, Norris GE, Rumball SV, Baker EN (1990). Apolactoferrin structure demonstrates ligand-induced conformational change in transferrins. Nature.

[28] Volkmann Niels (2013). The Joys and Perils of Flexible Fitting. Advances in Experimental Medicine and Biology.

[29] Ditzel L, Löwe J, Stock D, Stetter K-O, Huber H, Huber R, Steinbacher S (1998). Crystal structure of the thermosome, the archaeal chaperonin and homolog of CCT. Cell.

[30] Clare DK, Stagg S, Quispe J, Farr GW, Horwich AL, Saibil HR (2008). Multiple states of a nucleotide-bound group 2 chaperonin. Structure.

[31] Harris SP, Belknap B, Van Sciver RE, White HD, Galkin VE (2016). C0 and C1 N-terminal Ig domains of myosin binding protein C exert different effects on thin filament activation. Proc Natl Acad Sci.

[32] Wriggers W, He J (2015). Numerical geometry of map and model assessment. J Struct Biol.

[33] Kinghorn D. NAMD Molecular Dynamics Performance on NVIDIA GTX 1080 and 1070 GPU. https://www.pugetsystems.com/labs/hpc/NAMD-Molecular-Dynamics-Performance-on-NVIDIA-GTX-1080-and-1070-GPU-815/. Accessed: 26 Feb 2018.

[34] Kucukelbir A, Sigworth FJ, Tagare HD (2014). Quantifying the local resolution of cryo-EM density maps. Nat Methods.

[35] Afanasyev P, Seer-Linnemayr C, Ravelli RBG, Matadeen R, De Carlo S, Alewijnse B, Portugal RV, Pannu NS, Schatz M, van Heel M (2017). Single-particle cryo-EM using alignment by classification (ABC): the structure of *Lumbricus terrestris* haemoglobin. IUCrJ.

[36] Tan YZ, Baldwin PR, Davis JH, Williamson JR, Potter CS, Carragher B, Lyumkis D (2017). Addressing preferred specimen orientation in single-particle cryo-EM through tilting. Nat Methods.

[37] Hryc CF, Chen D-H, Afonine PV, Jakana J, Wang Z, Haase-Pettingell C, Jiang W, Adams PD, King JA, Schmid MF, Chiu W (2017). Accurate model annotation of a near-atomic resolution cryo-EM map. Proc Natl Acad Sci.

[38] Berman HM, Westbrook J, Feng Z, Gilliland G, Bhat TN, Weissig H, Shindyalov IN, Bourne PE (2000). The protein data bank. Nucleic Acids Res.

[39] Electron Microscopy Data Bank. http://www.emdatabank.org/.

[40] Jolley CC, Wells SA, Fromme P, Thorpe MF (2008). Fitting low-resolution cryo-EM maps of proteins using constrained geometric simulations. Biophys J.

[41] Wriggers W, Birmanns S (2001). Using *Situs* for flexible and rigid-body fitting of multi-resolution single-molecule data. J Struct Biol.

